# Radical Imagination: An Afrofuturism and Creative Aging Program for Black Women’s Brain Health and Wellness

**DOI:** 10.3390/ijerph22060875

**Published:** 2025-05-31

**Authors:** Tanisha G. Hill-Jarrett, Ashley J. Jackson, Alinda Amuiri, Gloria A. Aguirre

**Affiliations:** 1 Department of Neurology, Memory and Aging Center, University of California, San Francisco, CA 94143, USA; 2 Global Brain Health Institute, University of California, San Francisco, CA 94158, USA

**Keywords:** community engagement, Black women, Afrofuturism, creative aging, brain health, intersectionality, Radical Imagination

## Abstract

Intersectional oppression and invisibility are primary drivers of cognitive and mental health disparities that affect Black women’s wellness. Older Black women additionally experience compounding effects of ageism, which may place them at increased risk for a decline in cognitive functioning and mental wellness. To date, limited strengths-based, culturally relevant programming has focused on aging Black women. Fewer have incorporated Black women elders into conversations on Black liberation and the transformational change needed to create possible futures rooted in equity, healing, and health. This manuscript describes the inception and development of *Radical Imagination*, a creative aging program for Black women in the San Francisco Bay Area. Over ten weeks, 42 Black women (*M* age = 73.6, *SD* = 6.20; range: 58–85 years old) participated in the program, which incorporated brain and mental health education, art-making, storytelling, and photography. Grounded in principles of Afrofuturism and radical healing, participants explored past narratives of Black women and created a collective vision for a future that centers on Black women’s needs. Approximately 54.8% of participants attended more than one workshop. Upon program completion, exit surveys indicated that participants reported a moderate level of hopefulness about their ability to shape the future. Respondents reported overall satisfaction with the workshop series. We conclude with reflections on our process and recommendations for ways to support aging Black women using Afrofuturism and the arts.

## 1. Introduction

Systemic inequity is a primary driver of physical and mental health disparities, which disproportionately affect Black women’s wellness. Black women experience intersectional oppression (cross-cutting racism and sexism) [1,2,3] and intersectional invisibility (the erasure of Black women’s experiences and/or their contributions) [4,5]. These harms span multiple domains of influence and can be enacted at both the interpersonal and structural levels [6,7]. Growing evidence has linked these adverse social exposures to poorer mental [1,3,8,9] and physical health [2,10] outcomes among Black women. These exposures may accumulate over time, taxing Black women’s stress response system and causing them to “weather”—that is, to experience accelerated biological aging and decline in health compared to other racial-gender groups [11,12].

Older* Black women represent an especially vulnerable group, given their compounding experience of ageism [13,14]. (*: While “older” adulthood is typically defined as age 65 and older in the United States, we seek to extend our review and analysis to 50 years of age and above, as gendered and racialized health disparities that disproportionately impact Black women may emerge and become pronounced as early as midlife (e.g., see [15])). Existing research with Black women aged 50 years and older showed that their experiences of gendered racism, the simultaneous interpersonal experience of racism and sexism, are distinct from other racial-gender groups [16]. Older Black women’s experience of gendered racism is associated with more symptoms of depression, which, in turn, predicts self-reported concerns about cognitive function [17].

To adequately address existing threats to Black women’s mental and physical wellbeing, we must attend to both the historical and contemporary context of what it means to be a Black woman in the United States and offer a culturally relevant framework of care. This level of care and intervention incorporates an understanding of Black women’s lived experiences as well as their values and strengths. Leveraging the strengths of Black women counters the negative stereotypes and narratives that shape society’s perceptions and can mitigate biases in how researchers and healthcare professionals write, engage with, and provide care to Black women. A strengths-based approach divests from the “marginalized identity as deficit” narrative [18]. This, in turn, affirms and empowers Black women and places them as experts of their own experience [19]. Further, by explicitly naming the linked systems of oppression, it validates Black women’s reality and creates space for them to transcend the “matrix of domination” [20] (p. 18) and the oppressive boundaries placed on them.

The United Nations offers an example of tackling challenges of inequity and oppression through their Sustainable Development Goals [21]—a framework to address threats to health and wellbeing, gender equality, and sustainable communities. While focused on global challenges, important insights can be gleaned about ways to initiate local action and intervention. To date, limited intervention and community-based programming has focused on older Black women (50+ years old). A few exceptions exist, including the California Black Women’s Health Project and Sistahs Aging with Grace and Elegance. We seek to contribute to this exciting, growing body of work with the development and implementation of *Radical Imagination*: *A Dream Space for Black Women* (hereafter referred to as *Radical Imagination*). *Radical Imagination* is a community-centered, strengths-based, creative aging workshop series for older Black women. With its development, we provide novel contributions to existing discourse and programming efforts through our use of Afrofuturism and the arts as a praxis for not only brain and mental health but also aging Black women’s liberation and possible futures. *Radical Imagination* places Black women as central story writers of a reimagined future and explores new ways of Black women being in relation to their health and wellness. In the current manuscript, we present (1) the framework for the development of *Radical Imagination* and (2) preliminary findings from the implementation of the *Radical Imagination* pilot.

### 1.1. Framework

#### 1.1.1. Afrofuturism

*Radical Imagination* merges Afrofuturism with elements of a psychological framework of radical healing for People of Color and Indigenous individuals (POCI) [22]. Afrofuturism is an epistemology, artform, and “spatiotemporal consciousness” [23] that explores the African diaspora experience through alternate realities and futures using imagination, technology, and science fiction. While the term was originally coined by Mark Dery in his 1994 essay, *Black to the Future* [24], Afrofuturists and the practice of Afrofuturism have existed for centuries and is an intuitive and innate part of Black ancestral practices. Currently, there is no universal structure or definition that confines Afrofuturism neatly into a box, which is one of its many strengths. However, core components of Afrofuturism frequently include themes of (1) reclamation, (2) Black liberation, and (3) revisioning the past and predicting the future [25]. Afrofuturism counters the Western gaze and places Black people as the foremost storytellers of the future. This approach is advantageous as existing research acknowledges the benefit of Black people envisioning possible futures in which they are central [26].

A growing number of community-facing academics and practitioners employ community engagement methods that reject historical notions of marginalized communities being “subjects” of study and having little to offer [27]. These approaches to working within the community are extractive, cause harm, and contribute to mistrust [28,29,30]. Existing in the liminal space between the empirical and the imaginary, Afrofuturism is a critical method and praxis that resonates with the Black community, facilitates knowledge co-production, and advances society towards equity and Black liberation [31]. Envisioning Black women in the future free of struggle and safe in their bodies requires a radical reimagining of society as it exists today and aligns with Afrofuturistic thinking [32]. Additionally, Afrofuturism allows Black women to create counternarratives and counterstories that transcend racist and sexist tropes and challenge dominant ideologies [33]. The reclamation of Black woman imagery and discourse in fully actualized and nuanced ways dispels monolith beliefs, and this reclamation was an intentional element of *Radical Imagination*.

The final theme of Afrofuturism acknowledges alternative temporalities and the nonlinearity of time. This is important because Black Americans have been denied access to their pasts via the transatlantic slave trade and the intentional erasure of a history that predates enslavement. Black Americans were simultaneously forbidden access to the “temporal domain of the Western progressive future” [23] (pg. 20). This manifests in the present day as time inequity that disproportionally impacts Black Americans. Afrofuturism engages directly with the politics of time (Who is afforded time? Whose time is cut short? How does time equate to access and capital?) and, in turn, shifts the meaning, experience, and measurement of time [34,35]. Afrofuturists defy the purported sequential nature of time through a simultaneous forward and backward reaching process that “corrects the past and reshapes the future” towards equity [26] (p. 29). Afrofuturism is revolutionary and transformative and is directly linked to Black survival and prosperity [36,37]. Spanning both the personal and the political for Black women, it is with these considerations in mind that *Radical Imagination* is grounded.

#### 1.1.2. Radical Healing

Radical healing [22] is a psychological framework that recognizes the historical and contemporary experiences of racism faced by POCI. Radical healing provides space for POCI to resist oppressive systems while simultaneously moving toward societal transformation, collective wellness, and liberation. It is comprised of five evidence-based elements relevant to the wellness of POCI: (1) collectivism, (2) critical consciousness, (3) cultural authenticity and self-knowledge, (4) strength and resistance, and (5) radical hope. Radical healing is rooted in a social justice orientation and was developed to emphasize healing versus coping, an important distinction that advances POCI’s response to race-related stress and trauma from a reactionary to a proactive one [38]. Thus, the shift from Black women passively surviving oppression to actively resisting alongside others who share a similar history is a critical tenant of the framework and is an intentional focus of *Radical Imagination*. Importantly, the framework acknowledges the back-and-forth dialect of existing in the space of resistance against oppression and advancement toward liberation; as such, the process of acknowledging and imagining co-occur.

Radical healing additionally emphasizes collectivism and communal wellness. There is an intentional deviation from an individualistic, Western approach to intervention to one promoting community-level healing and wellness that is aligned with Black ways of being and relating [39]. This approach requires expansion in the belief of where and how healing can occur and is not limited to a medicalized clinical setting. Fittingly, *Radical Imagination* was hosted at a community center in a neighborhood where many of the workshop participants live, reside, and age.

Critical consciousness [40] is a component of radical healing that reflects the ability to reflect, ask difficult questions, recognize one’s agency, and take sociopolitical action. Cultural authenticity and self-knowledge, also components of this framework, encompass the appreciation of African ancestral ways of knowing and existing and maintaining pride in one’s Black identity. This includes acknowledging the importance of Afrofuturism and a decolonized imagination as critical precursors to liberation for POCI. Strength against oppressive systems is a necessary element of the radical healing framework. *Radical Imagination* participants considered what it meant to not only resist Western colonial violence but to commit to living a life of joy and abundance despite the state of society. We sought to embody radical hope: the belief that efforts towards change are not in vain and that a future rooted in equity can be actualized. Radical hope has been instrumental to the existence and livelihood of Black Americans [32] and can lessen the consequences of gendered racism experienced by Black women on their physical and mental health [41,42].

In summary, our program adopts both frameworks of Afrofuturism and radical healing and employs a strengths-based, culturally relevant approach where Black women can name and challenge systems of oppression in the present day (radical healing) while also having control over their pasts and futures (Afrofuturism). We approach this work from an ethic of love and care [43] and seek to engage with *Radical Imagination* participants in a way that disrupts power imbalances. Our goal is for transformation and healing justice—that is, for Black women to design and discover new ways of being in relation to themselves, to healing, and to their futures. We additionally seek to reassert Black women’s agency and self-determination as they completely govern *who* is seen and *how* they are seen.

#### 1.1.3. Creative Aging for Brain Health and Wellness

The arts enrich lives and create a unified language that transcends differences in culture, race, age, ability, socioeconomic status, and other aspects of lived experience. Participation in the arts is associated with numerous benefits to both health and wellbeing across the lifespan (e.g., see [44] for an overview). Creative aging is the participatory engagement in the arts and cultural activities to promote imagination and support older adults to age well [45,46]. Among older adults, engagement in arts-based practices is associated with reduced loneliness and increased interest in life [47], improved quality of life [48], maintenance of white matter integrity in brain regions associated with memory [49], better physical health [50], and reduction in depression symptoms [51]. The incorporation of Afrofuturism as a core form of artistic expression in *Radical Imagination* was therefore intentional; not only may Afrofuturism resonate with aging Black adults above and beyond “traditional” or mainstream creative aging approaches, but it may also be linked to positive cognitive health and wellness outcomes [32].

*Radical Imagination* was designed with the objective of supporting healthy cognitive aging. Healthy aging involves the maintenance of the cognitive, emotional, physical, and psychological health of adults living without dementia or significant cognitive impairment [52]. Participation in creative practices and the arts may contribute to healthy aging and brain health via the facilitation of neuroplasticity [53,54] and/or the maintenance of cognitive reserve [55,56]. Evidence exists highlighting the malleable nature of the brain as well as the presence of neuroplasticity in older age [57,58,59]. For aging Black women, the brain health benefits of creative practice are promising, given the increased risk of Alzheimer’s disease and related dementias (ADRD) in Black Americans [60,61] as well as women [62]. Creativity involves the use of cognitive operations that may contribute to healthy aging and brain health—namely, the ability to partake in divergent thinking, establish relationships between seemingly unrelated elements, and imagine future outcomes that have yet to exist. These core cognitive processes require cognitive flexibility, introspection, and the mental representation of possibility and may buffer against cognitive decline.

Lastly, *Radical Imagination* sought to use creative practices to contribute to community wellbeing, defined as the combination of social, economic, environmental, cultural, and political conditions identified by individuals in their communities as essential for them to flourish and fulfill their potential [63,64]. For some marginalized communities, wellbeing is encompassed by improved physical and mental health, community resilience and creative responses to racism and trauma, and civic capacity for structural change [65]. Community wellbeing also includes joy as well as preservation and celebration of Black culture [65], and *Radical Imagination* sought to introduce these components through participant narrative expression (photography, interviews, collages). *Radical Imagination* lastly aimed to promote social connectedness and cohesion, both of which are critical to collective action towards shared, communal goals and related to the United Nation’s Sustainable Development Goal #11 (to make cities more inclusive, safe, and resilient) [21].

## 2. Materials and Methods

### 2.1. Positionality Statements

The facilitator and co-facilitators of *Radical Imagination* are employees of the University of California San Francisco (UCSF) and participate in community outreach and engagement efforts within the UCSF Memory and Aging Center (MAC). When referencing *Radical Imagination* participants, we use the words “older” and “elder” interchangeably as the average age of the group was approximately 74 years, and within Black American culture, “elder” is often used as a term of respect and reverence. As a part of writing this manuscript, each of the authors engaged in critical self-reflexivity as a means of considering the ways their identities and lived experiences shape their approach and understanding of this work [66].

TGH-J: I am a Black American woman born and raised by Black women. My lived experiences are the lens through which I recognize and call out systems of oppression and develop strategies to dismantle them to serve the community. My Black womanhood affords a compassion and ability to relate to others on a human level as I see myself, and family members, in the *Radical Imagination* participants’ fight for visibility and wellness. I acknowledge that my education affords opportunity and access to spaces that also influence my worldview, including the way I engage in community-driven work. I recognize the age difference between myself and the participants, and I use this as a chance to learn from my elders and to unsettle power imbalances through collaboration and careful listening. As a person raised on the East Coast, I approach this work with a humble enthusiasm for learning about Bay Area culture and hope this work serves as an offering of appreciation to the Bayview community.

AJJ: Being a Black woman raised in the South of the United States, I am aware that my upbringing, personal experiences, and societal positioning have shaped my perspective and outlook in life. Through a lens deeply rooted in historical civil rights activism, cultural traditions, and faith-based practices, I aim to interact with the world with compassion, empathy, and advocacy. I recognize the historical and systematic injustices that specifically impact the Black community and support the importance of advocating for marginalized voices while fostering inclusivity in our magnificently diverse culture. As a Black woman, I’m honored to connect with the aging adults of the *Radical Imagination* community, and I appreciate the value of interacting with people from multiple generations. I recognize the contributions of older individuals that come from resilience and strength, and I aim to contribute to the remaining journey against oppression, racism, gender inequality, and ageism. I hope to continue positioning myself as a champion for the empowerment and wellbeing of our aging Black women. In standing with an understanding listening ear and supporting their need for a safe space, I want to continue encouraging them to view themselves with value and brilliance.

AA: As the co-facilitator of the *Radical Imagination* community workshop series, my positionality is shaped by my identity as a first-generation American-born Black woman with East African heritage. Growing up in the Bay Area, I’ve directly observed the acute challenges faced by Black women, fueling my commitment to addressing these issues within our community. My personal connection to these challenges underscores my dedication to working in spaces like *Radical Imagination*, where the focus is on uplifting and empowering older Black women. Centering participants’ unique stories and celebrating the resilience that defines their experiences is integral to fostering an intergenerational dialogue, bridging gaps, and fostering mutual understanding. While I’m relatively new to the realm of Afrofuturism, my enduring love for sci-fi serves as a wellspring of inspiration. I believe these workshops represent more than a response to identified disparities; they embody a proactive and creative step toward change.

GAA: I am a white, Cuban woman of mixed-race background who was born and raised in Miami, Florida. Growing up in a family of displaced immigrants, artists, and freedom fighters strongly influences my perspective and approach to grassroots, community-based equity work. I am privileged to have grown up in a majority-minority county of the United States, where the teachers, leaders, and protectors from my earliest experiences in life were often Black and Brown people. As a person who has lost the physical photos, notes, and keepsakes that hold the history of my family, I am deeply committed to preserving lived experiences and oral histories for future generations. I have been honored as the receiver of many stories and moments of wisdom from elders in my family and community, and I work to support the preservation and sharing of personal histories and lived wisdom through the arts, activism, and intentional allyship.

### 2.2. Participants and Setting

Participants of *Radical Imagination* included 42 self-identifying Black women (*M* age: 73.6, *SD*: 6.20; range: 58–85 years old who lived in a San Francisco Bay Area senior center and/or attended programming at the center. The center is in the Bayview neighborhood of San Francisco, California, and offers housing for eligible seniors. The UCSF community outreach team has an ongoing partnership with the senior center and has previously hosted brain health events at their facility. Participants of the program were identified through advertisements at the senior community center, brain health education talks at other centers (e.g., local YMCA), email listservs, and word-of-mouth referrals. *Radical Imagination* was also offered as part of Creative Minds, an arts for brain health collective developed at the UCSF MAC that provides other art workshops (e.g., photography, dance) and community programming. A total of 38.6% (*N* = 17) *Radical Imagination* participants were cross-enrolled in other Creative Minds programs. The workshop series received UCSF IRB approval (IRB # 20-32509) under the category of exempt research. Participants included in images additionally completed a separate video/photo consent form.

### 2.3. Program Description

*Radical Imagination* consisted of 10 weekly, two-hour workshops that were held between April and June 2023. Each week’s workshop was led by a facilitator (THJ) who is a licensed clinical neuropsychologist and has expertise in Black women’s cognitive aging, and a co-facilitator (AJ, AA) who has experience with Black/African American community engagement and research. The workshops were not “closed” to new enrollees, and there were no specific rules around attendance, although participants who attended all workshops received an additional gift of appreciation. While we approached this work with ideas in mind about the potential structure, we considered participants collaborators in the workshop series. We sought to be responsive and generate content that was inclusive of their ideas.

**Figure 1:**
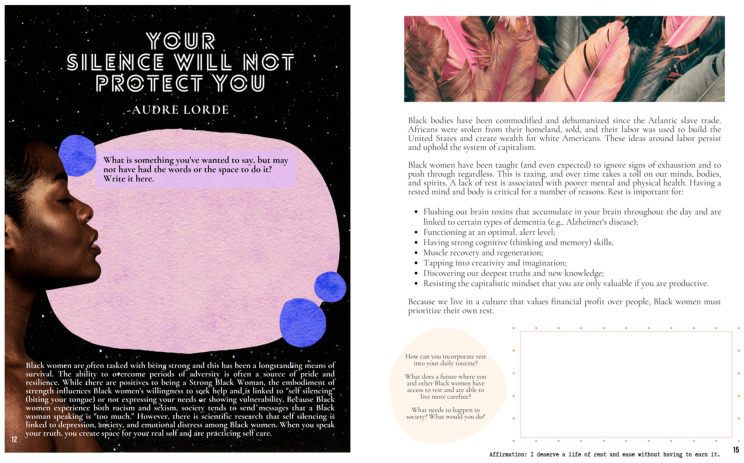
Sample pages from the *Radical Imagination* printed workbook used weekly by participants.

**Figure 2:**
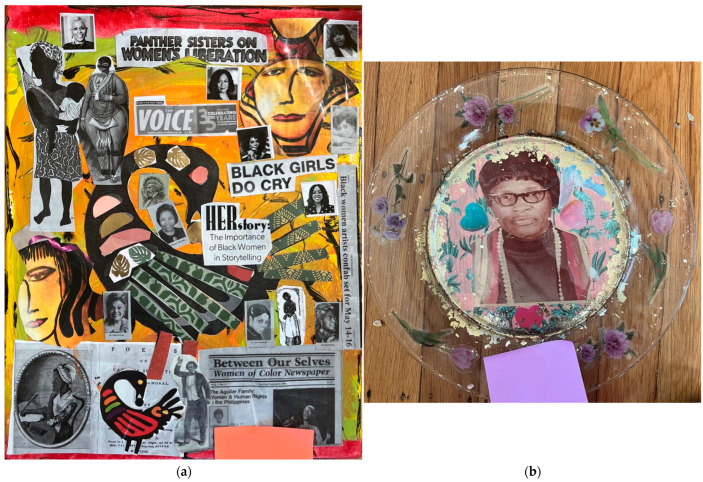
Artwork created by participants: (**a**) Collage on 11-inch × 17-inch canvas, photocopied newspaper, magazine clippings, cardstock, and acrylic; (**b**) gold foil, cardstock, and appliques on glass.

**Figure 3:**
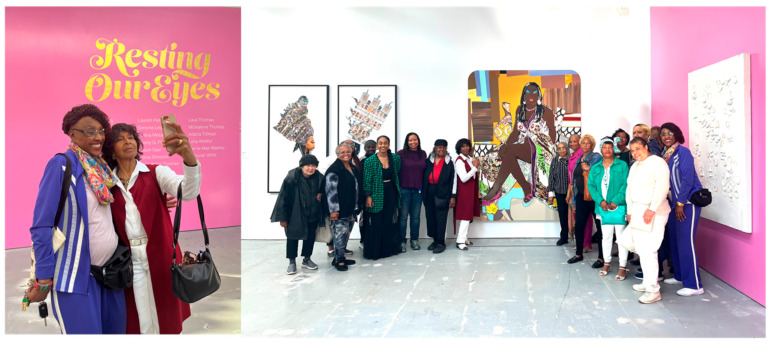
*Radical Imagination* participants visit the Institute of Contemporary Art San Francisco exhibit *Rest Our Eyes*.

All participants were provided a workbook (Figure 1) that corresponded to each week’s activities, presented an overview of relevant Afrofuturism and health concepts, and included guided prompts/reflection activities. As shown in Table 1, each workshop consisted of an Afrofuturism-themed didactic presented by the lead facilitator. The workshop additionally included guided group conversation and an art-related activity. The types of art activities ranged in scope from individual collage projects that incorporated the principles of Sankofa (i.e., a concept from the Akan people of Ghana that represents using wisdom from the past to inform the present and future) and honored Black women family/ancestors (Figure 2) to a group project that asked the women to work together to design new, future worlds in response to a prompt (e.g., What if the Black woman’s body was not subjected to scrutiny, sexualization, and criticism?). *Radical Imagination* invited one guest—a Black woman elder, creative writer, and artist—who facilitated a writing workshop. In addition to being producers of art, we were interested in *Radical Imagination* participants being art observers and having the opportunity to incorporate new perspectives into their art practice. Thus, Week 8 included a workshop plus a trip to a local museum to view an exhibition focused on Black women and rest (Figure 3). The final workshop in the series included a celebration, reflections, and presentation of gifts to all participants.

**Figure 4:**
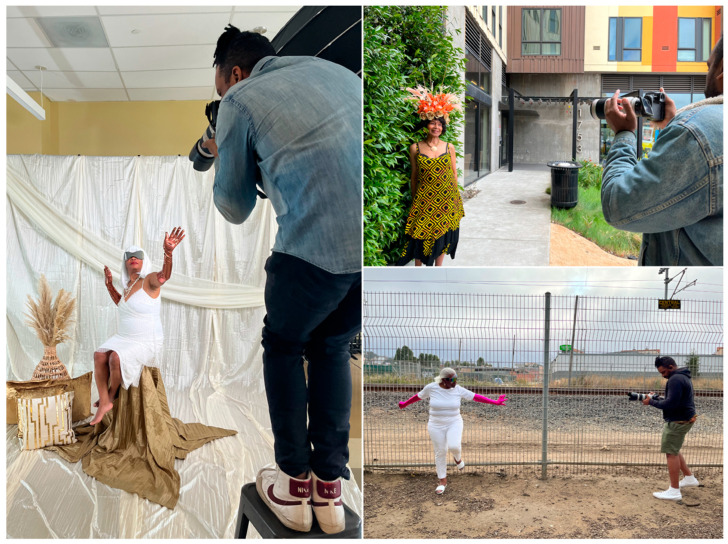
Behind the scenes of the photoshoot, *The Other Side of Time*, with photographer Austin James.

Thirteen *Radical Imagination* participants were also the voluntary subjects of an Afrofuturism portrait photoshoot (Figure 4). Titled *The Other Side of Time*, the photoshoot was a visual exploration of time inequities experienced by Black women and their reimagined futures. The final photos can be viewed in an online gallery [68]. As part of the experience, the lead facilitator completed audio interviews with each of the women about their vision for what it would mean to exist on the “other side”, where time, labor, and capital did not exist. The final artworks created by workshop participants and their Afrofuturism portraits were displayed in a Creative Minds community showcase held at a local arts and cultural center and were available on display for the public and participants’ family members.

### 2.4. Data Collection and Analyses

The stipulations of our existing grant funding required us to engage in programming and service delivery without the inclusion of a formal research component. This limits the forms of data that were collected during the implementation of *Radical Imagination*. Future iterations will include a collection of detailed demographics as well as a pre/post-survey design to evaluate participant benefits more directly. As a starting point, we present data from program implementation [69], including the results of our exit survey.

#### Post-Workshop Survey

A total of 18 *Radical Imagination* participants completed a paper-based survey for their exit interview (completed on the final day of the program), which sought to obtain feedback on the program content, aspects of the program that most interested participants, and suggestions for future program iterations. The survey consisted of 10 open-ended and 13 Likert-scale quantitative questions. An example open-ended question was: *What were the most important things you learned?* Example quantitative questions included: *I am hopeful about my ability to shape the future* with response options ranging from 1 = very untrue to 5 = very true and *How satisfied were you with the work-shop series?* with response options ranging from 1 = very dissatisfied to 5 = very satisfied. Demographic descriptives and Likert scale data (percentages) were analyzed using IBM SPSS 28.0. Salient quotes from open-ended responses were selected and agreed upon by the facilitator team. The quotes were thought to reflect and complement the quantitative data. We did not complete formal qualitative analyses of written participant responses as there was not enough detail for in-depth analysis. We plan to build upon these initial steps with future research using focus groups.

## 3. Results

### 3.1. Demographics

The average age of *Radical Imagination* participants was 73.6 years (*SD* = 6.20) at program entry. Participants ranged from 58 to 85 years old.

### 3.2. Retention

Workshop attendance for each week is shown in Table 1. Participation ranged from 14 to 26 attendees per workshop (*M* attendees = 18.8, *SD* = 3.39). On average, participants attended 4–5 workshops across the entire program (*M* = 4.45, *SD* = 3.71). A total of 54.8% (*N* = 23/42) of participants attended more than one *Radical Imagination* workshop. Of those who attended more than one workshop, repeat participants attended approximately seven workshops (*M* = 7.3, *SD* = 2.62) out of the entire series. A total of 11.9% (*N* = 5/42) of *Radical Imagination* participants attended all ten workshops.

### 3.3. Exit Survey

#### 3.3.1. Purpose for Attending

Open-ended, written responses to the survey indicated one of the main reasons for attending *Radical Imagination* included socializing/combating social isolation. One participant shared: “*I was made aware that this class was being offered and there was room enough for me. I don’t like being at home alone either”.* Similarly, participants shared they came out of “*curiosity and* [to] *engage with other women*” and because it “[*gave me*] *a reason to interact and share information*”. Another main reason for participation was that attendees were interested in learning about strategies to manage brain health and care for themselves. For instance, one participant stated she attended because she was interested in an “*activity pertaining to our health and memory*”. Others noted interest in “*learning more about the brain!”* and “*To learn more about how Black women can be more informed and active in the future*.” The final primary reason for attending *Radical Imagination* was to engage in art, Afrofuturism, and Black culture. Participants shared that they wanted “*to explore more ways to create art and learn new skills*”, “*wanted to know what Afrofuturism was all about and its true meaning*”, and “*learn more about Black people and how knowledgeable we are*”.

#### 3.3.2. Programming Takeaways

Quantitative survey results are presented in Table 2. With regard to interest in the programming, 81.3% of participants responded “very true”, and 12.5% responded “somewhat true” to find the topics of *Radical Imagination* interesting (0.1% responded “neutral”). A total of 87.5% of respondents indicated that they learned about topics that were important for Black women. Qualitative responses revealed that participants learned there is a commonality in some of the stressors faced by Black women ([“*We*] *all have some of the same problems*”), but they also recognized their agency in making change (“*What Black women do now will impact the future*”; “*The future is wide open for Black women if they want to be involved*”).

Participants shared that one of the most important things they learned through attending workshops was that they are creative and have artistic skills (“*My coloring and drawing skills are better than I thought*”; “*I did things I was proud of, like seeing myself doing different things with my mind*”). They also expressed a sense of importance around working toward a common goal with other women (“*To work together as a team with other women and feel as if I’m a part of something*”; “*I learned that if we work together we can achieve many positive things*”).

#### 3.3.3. Understanding and Application of Afrofuturism

A total of 47.1% of participants felt very confident in explaining Afrofuturism and its importance for Black women, and 29.4% were somewhat confident (23.5% were neutral). Respondents also rated their hopefulness about their ability to shape their future, with 61.1% indicating they are very hopeful in their ability and 33.3% feeling somewhat hopeful (5.6% were neutral). When asked what is in the future for Black women, respondents shared a sense of realistic optimism (“*We are going to make it. There is plenty more for us Black women, but we are going to make it*”; “*More opportunities than ever*.”; “*The future for Black women is very promising*”). When offering their projections, one woman identified strength as a necessary component of the future for Black women (“*To be strong Black women*”). Respondents recognized how integral Black women are to the future (“*There will be no future without us!*”) and Black women’s role as leaders (“*More leadership*”; “*More teaching others how to survive….and [be] happy*”). One respondent envisioned communal wellbeing as being part of the possible future (“*Greatness, maturity, compassion, and empowerment while being compassionate and empathetic towards community*”).

#### 3.3.4. Overall Interest in Radical Imagination

A total of 87.5% of respondents rated the art activities as very interesting, and 12.5% rated them as somewhat interesting. Regarding satisfaction with the workshop series, 72.2% and 22.2% were satisfied and very satisfied, respectively, and 94.4% expressed interest in attending another workshop in the future. Participants most enjoyed “*the freedom to create*.” Others appreciated the community of Black women formed, indicating that “[*The class*] *consisted of friendly and positive Black women*” and one participant found “*listening to women about my age expressing their feelings about various issues*” most enjoyable. Many respondents shared that they appreciated the facilitators (“*The teacher/trainer and staff….friendly*”; “[*They*] *were so great, loving, and understanding. Had much patience. Just great ladies*”). One respondent shared that they enjoyed challenging the stigma around aging, stating, “*Learning how we as older women came together and saw how our minds are very much together…it helped me with understanding that you*’*re never too old to learn*.”

#### 3.3.5. Recommendations

Common suggestions for improving future iterations of *Radical Imagination* were to include more brain health didactics and to provide a comprehensive list of workshop topics in advance. One participant suggested educational presentations on menopause. There was also interest in additional group excursions focused on the arts, such as attending concerts.

## 4. Discussion

We developed a community-based creative aging program for older Black women in San Francisco that incorporated components of the radical healing framework and Afrofuturism. Our preliminary data show strong acceptability. *Radical Imagination* participants found value in the program, and almost all (94%) expressed interest in participating in the next series of workshops. Our work contributes to the growing body of creative aging programs, with a focus on examining and dismantling social inequality that disproportionately impacts aging Black women. One of our objectives was to facilitate deep, critical inquiry into how systems of power and privilege impact whose stories get told and what voices are valued in conversations surrounding the future of society. “To be in the margin is to be part of the whole but outside the main body” [70] (p. 20), and *Radical Imagination* sought to map the experiences and possible futures of those at the margins.

Overall, the workshop sizes were moderate in terms of the number of attendees. Participation retention was 54.8%, with the average number of workshops attended ranging from four to five workshops out of the ten workshop series. Among those who were repeat *Radical Imagination* attendees, they attended 75% of the workshops on average, and there were five women who attended every workshop. These findings reflect the open workshop structure, which allowed participants to flexibly attend as they were available while also maintaining a “core” group of participants. There are both pros and cons that come with this approach to program design. This flexible approach affords increased accessibility, making the information contained within the workshops available to a larger group of people with varied perspectives. It also allowed for more expansive social networking. A flexible workshop structure may also be a more culturally responsive approach to community engagement for a demographic group that has experienced trauma and racism from university and medical institutions, as well as barriers to participation in aging-focused research [71,72]. Conversely, having the workshop series closed to a set number of enrollees facilitates more targeted instruction, deeper learning, and focused, intimate interactions. Future iterations of *Radical Imagination* should examine differences in the long-term impact of programming that prioritizes social reach versus depth.

**Figure 5:**
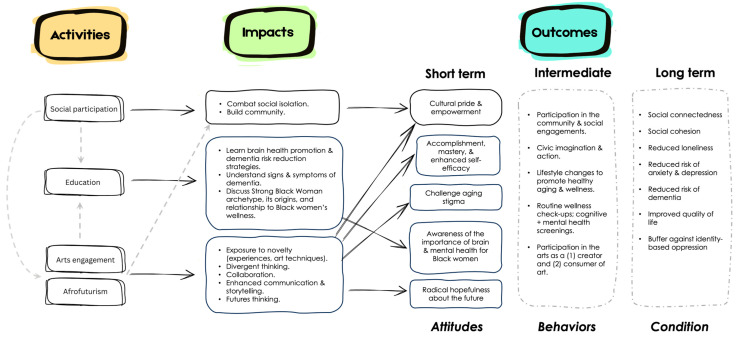
Logic model of *Radical Imagination* activities, impacts, and outcomes resulting from exit interview survey analysis. Solid arrows indicate relationships that were shared by participants in the exit interview and dashed arrows reflect hypothesized relationships. Short-term outcomes reflect proximal relationships that were articulated by participants in the exit interview; intermediate and long-term outcomes (dashed) are hypothesized.

Based on responses to the exit interview, there were several key areas of impact identified by participants. The areas of impact and their hypothesized relationships to short-, intermediate-, and long-term health and wellness outcomes are shown in the resulting Logic Model (Figure 5). Participants shared that one of the main reasons for participation in *Radical Imagination* was to counter social isolation and connect with other women. Social isolation is defined as the objective physical separation and lack of social contact between an individual and others. Older adults have a high risk of social isolation due to age-related changes in health and mobility [73], transportation limitations [74], loss of family and friends [75], and living alone [74,76]. A large body of research also suggests that social isolation is associated with dementia [77], lower quality of life [78], and mortality [79]. While not formally assessed, it is possible that *Radical Imagination* also addressed loneliness, a separate but related concept that speaks to the subjective experience of being alone, disconnected, and lacking meaningful social relationships. Loneliness is associated with lower cognitive functioning [80] and accelerated cognitive decline [81]. Participation in the arts and creative aging programs like *Radical Imagination* may counter loneliness and social isolation by promoting social connection [82] and social embeddedness within the community, creating a sense of belonging and enhancing a sense of contribution to society [83]. *Radical Imagination* participants also recognized the importance of working together toward a common objective via several of the activities, which may have also created a sense of closeness, community, and belonging.

Another point of participant feedback was appreciation of the arts and Afrofuturism. While some of the participants were initially skeptical of their artistic ability, many were pleasantly surprised by their creativity and final artworks. It is possible that through the process of creation, *Radical Imagination* participants experienced a sense of mastery and bolstered self-efficacy, which have positive benefits for mental health and health behaviors [84] and prospective memory [85]. There is also increasing recognition of how engagement in the arts is a health behavior that addresses mental health inequities [44,86], promotes healthy cognitive aging [87], and amplifies underrepresented voices. Individuals who participate in arts and education activities have lower negative affect and overall higher life satisfaction [88]. Afrofuturism intersects with the arts, and therefore, it likely affords the same health benefits as arts engagement. Additionally, Afrofuturism is thought to provide agency and freedom, a method of historical and speculative inquiry, and a culturally relevant language to express experiences of oppression but also radical hope. These additional components are critical and appeared to resonate with the Black women participants of *Radical Imagination* as a majority expressed hopefulness around the future, which also has positive health implications, particularly among older adults [89].

Black women are frequently excluded from visual imagery, dialogue, and rhetoric around science, speculative fiction, and projections of the future [90,91]. Both race and gender intersect with age to create a unique social space of exclusion for Black women elders. The gendered racial stereotypes that Black women experience (e.g., the Black woman as a caregiver or Mammy) [92] may become even more exacerbated as Black women age. In *Radical Imagination*, we intentionally designed activities like the photoshoot to showcase a Black aesthetic that counters the Western imagination and standards of beauty. Through our art practice, we also sought to invoke the memories of Black women’s ancestors who expanded beyond monolithic notions of Black womanhood. This intentional approach to Afrofuturism and art-making may contribute to cultural pride and empowerment at the individual and collective level [93] as well as identity centrality, which has served as protective factors against identity-based discrimination [2,94,95].

Regarding the practice and application of Afrofuturism, participants expressed a realistic optimism about their ability to shape the future and a general acknowledgment of their shared struggles for liberation. With that, many women also acknowledged Black women’s agency and leadership in enacting change. Participants expressed the belief that Black women will be an integral part of any future. In these conversations, there was general recognition amongst participants that Black women need to be strong, and there was an articulated sense of pride associated with strength. However, in our conversations as a group (that were not captured as part of the exit interviews), some also shared experiences of feeling tired from having to constantly be the strong one always in control. These sentiments reflect the Strong Black Woman schema: an archetype of Black womanhood consisting of unwavering emotional strength and suppression, self-sacrifice, prioritization of others’ needs (often at the expense of one’s own), and independence. The Strong Black Woman schema exists as a paradox in that strength and self-reliance are protective against the harms of intersecting structural oppressions [96,97] and a source of pride for many Black women [98], but these characteristics are also associated with emotional suppression [99], high drive to succeed, self-reliance, and the tendency to put the needs of others before oneself [100], which negatively impact both physical and psychological health [99,100,101,102,103,104].

Black women are socialized to be strong from childhood [105,106], and this strength is reinforced across the lifespan [107]. Current research suggests middle- and older-aged Black women more strongly endorsed Strong Black womanhood compared to younger adults, and older Black women did not recognize the schema’s influences on their health [108]. As matriarchs of their family, elder Black women uphold the mask of strength for decades, and this may take a toll over time. More attention is needed on ways to support elder Black women in envisioning both present and future versions of themselves in ways that maintain aspects of strong Black womanhood that bolster pride, sense self-esteem, and resilience while also minimizing aspects of the schema that lead to negative stress-coping and limited help seeking behaviors. Afrofuturism offers an innovative avenue of exploration and reimagining of topics like pleasure, ease, and vulnerability—elements of femininity, but also strength, that Black women have traditionally been denied access to [109,110].

Interestingly, one *Radical Imagination* participant was surprised by how their participation defied personal stigma and stereotypes. The research shows that individuals from disadvantaged backgrounds are more likely to be stigmatized than those from privileged groups [111], and internalized stigma can negatively influence self-perception. Another potential proximal outcome of *Radical Imagination* is that through achievement, mastery, and engagement in novel activities, they were able to challenge perceptions of aging and reduce internalized stigma (Figure 5). This may have beneficial downstream impacts on health as self-perception of aging is associated with social and cognitive engagement later in life [112].

It was our intention to celebrate aging. Often, elders are excluded from conversations about the future with preconceived notions that they may not have relevant ideas, which are steeped in ageist beliefs. Age and its associated wisdom was considered an offering to the conversations and ideations that occurred as part of *Radical Imagination* workshops. We considered participants “walking ancestors” [113] whose collection of knowledge and lived experience contributes to the shaping and scaffolding of tomorrows. Beyond their wealth of knowledge amassed throughout the life course, Black women elders hold an essential role as cultural custodians [114]; *Radical Imagination* sought to honor this important role and to expand it by having participants posit what Black American culture may look like centuries from now.

The final component of feedback gleaned from the exit survey was that *Radical Imagination* participants were motivated by the opportunity to learn about brain health and Black women’s wellness. Participant feedback indicated a desire for more integration of education into the programming. In our reflection on the educational component, PowerPoint presentation may be an effective modality for brief dissemination of information, but conversation and the opportunity to share in a group format maintained engagement and encouraged curiosity. Through this approach, there was also an opportunity to disrupt artificial educational hierarchies/imbalances as some *Radical Imagination* participants were once caregivers to family members who had dementia and had significant lived experience to contribute to the group. There also appeared to be a higher level of receptivity when information was presented in a game format (e.g., a prize was offered to the person who recalled the most information presented; brain health concepts were taught through an interactive game). How these different educational modalities differ in relation to information retention and implementation of health promotion/preventative behaviors warrants further investigation.

### Limitations and Future Directions

This work should be considered with some limitations in mind. The first is that our workshop series was not structured as a pretest/posttest design, and data were only collected upon program completion. The lack of baseline data limits the ability to draw conclusions about the benefits of the workshop series as an intervention. Ideally, future iterations of *Radical Imagination* would utilize a randomized control trial design, which includes a control group so that inferences regarding causality can be made. Secondly, our sample was focused on Black women living in a specific neighborhood of the San Francisco Bay Area. While this allowed us to tailor the program to their unique needs, the program activities and their potential benefits may not be fully generalizable to Black women elders in other regions of the United States and beyond. Additionally, our “open group” format may also impact the ability of the workshop and topics to build upon themselves, although many of the *Radical Imagination* weekly activities were designed to be independent of one another. This also may influence the survey responses, as not every person who attended a workshop completed feedback, which may also skew findings and influence interpretability. Feedback from program participants highlighted the complexity of the facilitator balancing the amount of brain health education presented with arts-based activities. While almost all survey respondents expressed interest in participating in future offerings *of Radical Imagination*, workshops should better integrate art with science and health.

With these factors in mind, there are several exciting avenues for future exploration. We viewed the Black women participants as co-creators of knowledge throughout this process, but there was also a structure from the outset that distinguished the role of the facilitator versus the participant. It would be worthwhile to explore alternative models, such as one where a community member serves in a co-facilitator role. Additional offshoots of the program may be worth considering, particularly as it relates to sharing brain health education amongst other community members who may not participate in the center’s activities (e.g., train the trainer models). As was mentioned by participants in their feedback, future work should include more health education. Perhaps this could be explored through not only the women being recipients of health education but also champions of it sharing education with the larger community. The act of Black women projecting themselves into the future is a revolutionary act, one that likely has brain health benefits given the cognitive processes involved, including imagination [115,116]. Future work is needed to more systematically examine the effect that adopting and or/maintaining a futuristic orientation and belief system has on cognitive aging and brain health outcomes. Black people have long practiced Afrofuturism as a way of being and intuiting, and their existence in the present day, despite tremendous adversity, is evidence of its benefits. We do not seek to attempt to impose Western forms of measurement onto Afrofuturism or box it in so that it is neatly measurable and “empirical”. However, the application of Afrofuturism as a praxis for liberation and brain health is certainly worth pursuing within academic medical environments as well as community contexts. Others are carefully considering how Afrofuturism is a methodology that creates an unflattening of the knowledge production process [117]. It can be adopted as an approach to amplify the impact of inequities and generate solutions to address them [31]. This work should be extended so that not only do those engaged in Afrofuturistic practices reap the potential brain health and wellness benefits but so that it is applied as a method of shaping equitable brain health futures.

## 5. Conclusions

*Radical Imagination* is an innovative program that combines principles of Afrofuturism, radical healing, and creative aging to support the health and wellness of older Black women. To our knowledge, this program is the first of its kind. *Radical Imagination* unflattens the knowledge production process, disrupts power imbalances (between the researcher and “researched”), and centers Black women elders’ visions for the future. Preliminary evidence suggests potential program impacts on participants’ social engagement, community building, cultural pride and empowerment, radical hopefulness, aging stigma, and brain health awareness. This work underscores the importance of using strengths-based, culturally responsive approaches to community engagement and community interventions that seek to reduce health disparities. Equity and social change can only be actualized when the needs and dreams of those at the margins are a central part of the solution.

## Figures and Tables

**Table 1:** Ten-week overview of each Radical Imagination workshop, attendance, learning objectives, and corresponding activities.

Workshop (Number of Attendees)	Purpose and Overview	Activities
1: Welcome and orientation (*n* = 21)	Build rapport/meet one another/begin developing group cohesion Provide an overview of Afrofuturism and the workshop series Introduce participants to brain health and mental health concepts (e.g., Strong Black Woman archetype); discuss the importance of wellness for Black women	Individual introductions and purpose for participation Review learning objectives Set “SMART” group goals Picnic working memory activity
2: Sankofa (“Go back to the past and bring forward that which is useful.”) (*n* = 26)	Review Sankofa concept Discuss how the past, present, and future are interconnected. Consider: What seeds for the future do we want to plant in the present?	Canvas collage activity using historical newspaper clippings and Black woman imagery
3: Legacy (*n* = 21)	Honor Black women throughout the ages Reflect on what each person brings to the group and what impression they want to leave on world Discuss how legacy is a component of storytelling and Black oral history tradition	Plate photo transfer project Four quadrants activity
4: Affirmations from the future (*n* = 17)	Provide overview of gendered racism, stress, and physiological consequences (HPA-axis, accelerated aging, weathering) Review the power affirmations and positive health effects	Affirmations worksheet Record for group affirmation mixtape
5: Black woman utopias (*n* = 17)	Compare/contrast utopias vs. dystopias Conversation around society’s trajectory Co-design a utopian world based on prompts given (e.g., What does a society full of safety for Black women look like 1000 years from now?) Discuss the concept of equity and conditions in society that would need to change to achieve equity; what are local issues, and what would need to change?	Complete utopia worksheet Group collaboration; different prompts requiring them to build a new utopian world at a randomly assigned number of years into the future Group presentations of worlds
6: The art of adornment (*n* = 18)	Discuss depictions/imagery of Black women in media Describe links between adornment and Black American tradition Review the history of cowrie shell and its significance in Black culture	Creation of headpiece/crown
7: Brain health education and common misconceptions (*n* = 17)	Present didactic on Alzheimer’s disease and related dementias (ADRD) in the Black community Debunk common misconceptions about aging and dementia Discuss advocating for self when navigating the healthcare system	Brain health true/false game Afrofuturism themed BINGO
8a: Shaping possible futures (*n* = 20)	Explore individual beliefs about the ability to impact the future; what influences belief systems? Collaborate with other participants to write a possible future	Exquisite corpse group poem Mixed media art
8b: We will rest (*n* = 14)	Review the relationship between sleep and brain health Discuss rest as reparations and the work of the Nap Ministry [67]	Museum trip
9: Celebration and reflection (*n* = 17)	Explore final group values Participants reflect and provide feedback for future iterations of *Radical Imagination*	Slide show of photo memories Presentation of gifts Food and music
10: Photoshoot: *The Other Side of Time* (*n* = 13)	Explore how time inequity disproportionately impacts Black women Construct a counternarrative that defies ideas around time, labor, and capital	Styled photoshoot Qualitative interviews

**Table 2:** Radical Imagination exit survey results (*n* = 18). Values reflect the percentage of participants that endorsed each response.

Question	Very Untrue	Somewhat Untrue	Neutral	Somewhat True	Very True
The topics discussed were interesting.	0.00	0.00	6.30	12.5	81.3
I learned about topics important for Black women.	0.00	0.00	12.5	6.30	81.3
I feel confident explaining Afrofuturism and its importance for Black women.	0.00	0.00	23.5	29.4	47.1
I am hopeful about my ability to shape the future.	0.00	0.00	5.60	33.3	61.1
The art activities in each class were interesting.	0.00	0.00	0.00	12.5	87.5
I am satisfied with the workshop series.	0.00	5.60	0.00	22.2	72.2
I would attend another workshop in the future.	0.00	0.00	5.60	11.1	83.3

## Data Availability

The data are not available because we do not have permission from participants to share this information outside of our research group.
